# Supplementary material for: Crawling and Gliding: A Computational Model for Shape-Driven Cell Migration
Source: PLoS Comput Biol. 2015 Oct 21;11(10):e1004280. doi: 10.1371/journal.pcbi.1004280 (PMC4619082; doi:10.1371/journal.pcbi.1004280)
Supplement: S1 Code — (ZIP) [file pcbi.1004280.s012.zip › release/tst/doc/html/output_8h_source.html]

Tissue Simulation Toolkit: output.h Source File


Tissue Simulation Toolkit
 0.1.4.1


- Main Page
- Namespaces
- Classes
- Files

- File List
- File Members

output.h

Go to the documentation of this file.

1 /\*

2

3 Copyright 1996-2006 Roeland Merks

4

5 This file is part of Tissue Simulation Toolkit.

6

7 Tissue Simulation Toolkit is free software; you can redistribute

8 it and/or modify it under the terms of the GNU General Public

9 License as published by the Free Software Foundation; either

10 version 2 of the License, or (at your option) any later version.

11

12 Tissue Simulation Toolkit is distributed in the hope that it will

13 be useful, but WITHOUT ANY WARRANTY; without even the implied

14 warranty of MERCHANTABILITY or FITNESS FOR A PARTICULAR PURPOSE.

15 See the GNU General Public License for more details.

16

17 You should have received a copy of the GNU General Public License

18 along with Tissue Simulation Toolkit; if not, write to the Free

19 Software Foundation, Inc., 51 Franklin St, Fifth Floor, Boston, MA

20 02110-1301 USA

21

22 \*/

23 #ifndef OUTPUT\_H\_

24 #define OUTPUT\_H\_

25

26 #ifdef \_\_cplusplus

27 extern "C" {

28 #endif

29

30 int OpenFileAndCheckExistance(FILE \*\*fp,const char \*fname,char \*ftype);

31 int YesNoP(const char \*message);

32 FILE \*OpenWriteFile(const char \*filename);

33 FILE \*OpenGZippedWriteFile(const char \*filename);

34 FILE \*OpenReadFile(const char \*filename);

35 char \*ReadLine(FILE \*fp);

36 void CheckFile(FILE \*fp);

37 int FileExistsP(const char \*fname);

38 char \*Chext(char \*filename);

39 void MakeDir(const char \*dirname);

40 bool CanWeWriteP(char \*filename);

41

42 #ifdef \_\_cplusplus

43 }

44 #endif

45

46

47 #define MESS\_BUF\_SIZE 160

48 #ifndef FALSE

49 #define FALSE 0

50 #define TRUE 1

51 #endif

52

53 #endif

CanWeWriteP

bool CanWeWriteP(char \*filename)

OpenReadFile

FILE \* OpenReadFile(const char \*filename)

**Definition:** output.cpp:139

MakeDir

void MakeDir(const char \*dirname)

CheckFile

void CheckFile(FILE \*fp)

**Definition:** output.cpp:226

YesNoP

int YesNoP(const char \*message)

**Definition:** misc.cpp:120

Chext

char \* Chext(char \*filename)

**Definition:** output.cpp:235

OpenWriteFile

FILE \* OpenWriteFile(const char \*filename)

**Definition:** output.cpp:105

FileExistsP

int FileExistsP(const char \*fname)

**Definition:** output.cpp:47

OpenFileAndCheckExistance

int OpenFileAndCheckExistance(FILE \*\*fp, const char \*fname, char \*ftype)

**Definition:** output.cpp:35

OpenGZippedWriteFile

FILE \* OpenGZippedWriteFile(const char \*filename)

ReadLine

char \* ReadLine(FILE \*fp)

**Definition:** output.cpp:156


---

Generated on Thu Aug 14 2014 22:04:01 for Tissue Simulation Toolkit by  

 1.8.6
